# Towards the introduction of pneumococcal conjugate vaccines in Bhutan: A cost-utility analysis to determine the optimal policy option

**DOI:** 10.1016/j.vaccine.2018.02.048

**Published:** 2018-03-20

**Authors:** Kinley Dorji, Sonam Phuntsho, Pempa, Suthasinee Kumluang, Sarayuth Khuntha, Wantanee Kulpeng, Sneha Rajbhandari, Yot Teerawattananon

**Affiliations:** a Essential Medicine & Technology Division, Ministry of Health, PO Box 726, Thimphu, Bhutan; b Policy & Planning Division, Ministry of Health, PO Box 726, Thimphu, Bhutan; c Health Intervention and Technology Assessment Program, 6th Floor, 6th Building, Department of Health, Ministry of Public Health, Tiwanon Rd., Muang, Nonthaburi 11000, Thailand

**Keywords:** Cost-utility analysis, Pneumococcal conjugate vaccines, PCV, Human resources for health, Bhutan

## Abstract

**Background:**

Due to competing health priorities and limited resources, many low-income countries, even those with a high disease burden, are not able to introduce pneumococcal conjugate vaccines.

**Objective:**

To determine the cost-utility of 10- and 13-valent pneumococcal conjugate vaccines (PCV10 and PCV13) compared to no vaccination in Bhutan.

**Methods:**

A model-based cost-utility analysis was performed in the Bhutanese context using a government perspective. A Markov simulation model with one-year cycle length was used to estimate the costs and outcomes of three options: PCV10, PCV13 and no PCV programmes for a lifetime horizon. A discount rate of 3% per annum was applied. Results are presented using an incremental cost-effectiveness ratio (ICER) in United State Dollar per quality-adjusted life year (QALY) gained (USD 1 = Ngultrum 65). A one-way sensitivity analysis and a probabilistic sensitivity analysis were conducted to assess uncertainty.

**Results:**

Compared to no vaccination, PCV10 and PCV13 gained 0.0006 and 0.0007 QALYs with additional lifetime costs of USD 0.02 and USD 0.03 per person, respectively. PCV10 and PCV13 generated ICERs of USD 36 and USD 40 per QALY gained compared to no vaccination. In addition, PCV13 produced an ICER of USD 92 compared with PCV10. When including PCV into the Expanded Programme on Immunization, the total 5-year budgetary requirement is anticipated to increase to USD. 3.77 million for PCV10 and USD 3.75 million for PCV13. Moreover, the full-time equivalent (FTE) of one health assistant would increase by 2.0 per year while the FTE of other health workers can be reduced each year, particularly of specialist (0.6–1.1 FTE) and nurse (1–1.6 FTE).

**Conclusion:**

At the suggested threshold of 1xGDP per capita equivalent to USD 2708, both PCVs are cost-effective in Bhutan and we recommend that they be included in the routine immunization programme.

## Highlights

• PCV13 and PCV10 are both cost-effective in Bhutan.
• PCV13 yields better health outcomes and slightly more cost than PCV10.
• Introducing PCV would reduce the workload of scarce medical specialists in Bhutan.

## 1 Introduction

Pneumococcal disease is an infection caused by the *Streptococcus pneumoniae* ***(****S. pneumoniae****)*** bacteria [1]. This infection can result in meningitis, bacteraemia, pneumonia, and acute otitis media (AOM). Pneumonia has been a leading cause of child morbidity and mortality globally, accounting for about 1.6 million deaths annually in children under five years of age [2]. Incidence and mortality rates are high in low-income countries with the majority of pneumococcal deaths occurring in Africa and Asia. To combat pneumococcal disease, various types of vaccines have been developed. Currently, 10-valent pneumococcal conjugate vaccine **(**PCV10**)** and 13-valent pneumococcal conjugate vaccine **(**PCV13**)** are available on the market, which have proven to be safe and efficacious against *S. Pneumoniae* [3]. The high burden of pneumococcal disease in developing countries has led to global efforts in expanding the access to vaccines in these regions.

Bhutan is a lower middle-income country located in South Asia where pneumococcal infections remain a major cause of morbidity and mortality among young children. In 2015, there were 349 cases of meningitis and 10,891 cases of pneumonia [4]. The case fatality rate of meningitis due to *S. pneumoniae* was 7%. The Bhutanese government spends a large amount of health budget on the treatment of pneumococcal disease, which also have high societal costs. The National Committee for Immunization Practice, an independent technical advisory committee to advice and guide the Ministry of Health, Bhutan, recommended the introduction of pneumococcal conjugate vaccines in the country, that was needed to conduct an economic evaluation.

The Expanded Programme on Immunization was first launched in Bhutan in 1979. The programme maintains high coverage with a routine immunization package. Bhutan has graduated from Gavi, the Vaccine Alliance's support in 2016 as its economic classification status has changed from a low income to lower-middle income country. As such, considerations around value for money and financial sustainability of the routine vaccination programme are of critical importance to Bhutan because introducing new vaccines poses a direct and long-term financial burden to the government. Prior to the introduction of the vaccines, the World Health Organization's Strategic Advisory Group of Experts on Immunization recommends all member states to conduct a systematic decision making process based on review of evidence from cost-effectiveness analysis and budget impact analysis studies [5]. Subsequently, the High Level Committee, the highest decision making body in the Ministry of Health, Bhutan, directed to conduct a cost-utility analysis of PCVs to inform policy decision and vaccine implementation.

In response to policy makers in Bhutan, this study aimed to determine the costs, outcomes and cost-effectiveness of the introduction of PCV10 and PCV13 compared to a no vaccination policy. In addition, the study also determined the feasibility of the vaccination policy by assessing human resource on health impact and 5-year budget impact for introduction of pneumococcal conjugate vaccines.

## 2 Methods

A model-based cost-utility analysis (CUA) was performed and government perspective was considered for the study. A Markov model was constructed to estimate the costs and health outcomes of infants using a lifetime horizon with a 3% discount rate per annum. Each of the three policy options, namely no vaccination, PCV10, and PCV13 were evaluated. The vaccine schedule was two-dose primary series at 2 months and 4 months, and one booster at 12 months of age (2 + 1 schedule). Health outcomes measured included number of pneumococcal episodes averted, number of deaths prevented due to the vaccination programme and quality-adjusted life year (QALY). The incremental cost-effectiveness ratio (ICER) was presented as cost in United State Dollar (USD) per QALY gained (USD 1 = Bhutanese Ngultrum 65).

### 2.1 Model structure and assumptions

**Fig. 1:**
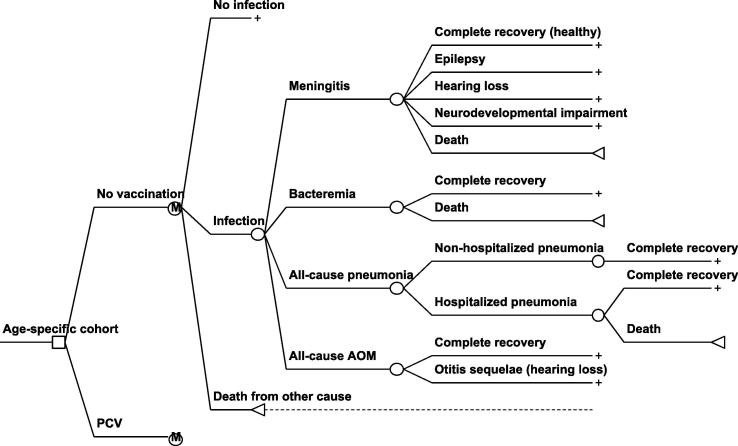
Age-stratified economic model to represent associated health states for vaccinated and unvaccinated populations.

A Markov model with one-year length of cycle was adapted from a Thai study [6] to compare costs and health outcomes of PCVs with no PCV programme for each age-specified cohort as seen in Fig. 1. The model assumes that vaccinated and unvaccinated individuals can experience each three different health events: no infection, S. pneumoniae infection, and death from all other causes. *S. pneumoniae* infection leads to four main diseases including meningitis, bacteraemia, pneumonia and AOM. Moreover, meningitis is associated with sequelae such as epilepsy, hearing loss, and neurodevelopment impairment, as well as AOM causes hearing loss. A cost-utility of vaccination programme for a single hypothetical birth cohort born in 2016 was simulated for a lifetime horizon which means that all individuals in this group are followed until transition to the death state at a maximum of 100 years of age. For a budget impact analysis, vaccination on hypothetical cohorts of infants born during 2016–2020 were examined over a period of five years. Two vaccine scenarios i.e. with and without indirect effects were modeled.

### 2.2 Model input parameters

#### 2.2.1 Epidemiological data

The disease incidence rates of meningitis, bacteraemia, pneumonia, and acute otitis media were derived from the Annual Health Bulletin 2016 [4] and through literature reviews [7], [8]. The incidence rates of pneumococcal bacteraemia and sequelae were transferred from Thai studies [9], [10] as these data were not available in Bhutan and were explored by distribution of age as seen from Supplementary Table 1. Mortality rates and probability of developing sequelae were derived from literature reviews [11] and illustrated in Table 1.

**Table 1:** Model input parameters.

Parameters	Distribution	Mean	SE	Ref.
Pneumococcal meningitis incidence per 100,000^a^				[4], [7], [8]
Aged <1	Beta	131.69	15.37	
Aged 1–4	Beta	12.85	2.39	
Aged 5–14	Beta	7.55	1.07	
Aged 15–19	Beta	4.60	0.36	
Aged 20–49	Beta	4.60	0.88	
Aged 50–64	Beta	4.11	1.33	
Aged ≥65	Beta	5.32	15.37	
Pneumococcal bacteraemia incidence per 100,000				[10]
Aged <4	Beta	11.1	1.84	
Aged 5–19	Beta	1.4	0.33	
Aged 20–49	Beta	1.9	0.28	
Aged 50–64	Beta	4.6	0.79	
Aged ≥65	Beta	13.6	2.02	
All-cause pneumonia incidence per 100,000				[4]
Aged <1	Beta	19117.07	340.31	
Aged 1–4	Beta	7373.52	109.60	
Aged 5–14	Beta	708.28	20.21	
Aged 15–19	Beta	277.82	8.32	
Aged 20–49	Beta	277.82	36.02	
Aged 50–64	Beta	809.48	61.77	
Aged ≥65	Beta	1339.13	340.31	
All-cause AOM incidence per 100,000				[4]
Aged <1	Beta	5267.77	188.32	
Aged 1–4	Beta	3000.88	70.83	
Aged 5–14	Beta	2395.54	37.01	
Aged 15–19	Beta	1507.20	19.31	
Aged 20–49	Beta	1507.20	49.23	
Aged 50–64	Beta	1517.35	57.39	
Aged ≥65	Beta	1155.00	188.32	
PCV13 efficacy^b^				
Vaccine-type IPD (3 + 1)	Beta	89.00%	3.57%	[12]
Radiologically-confirmed pneumonia (3 + 1)	Beta	25.50%	8.72%	[13]
All-cause AOM (3 + 1)	Beta	6.00%	1.53%	[12]
PCV10 efficacy				
Vaccine-type IPD	Beta	92.00%	10.71%	[14]
Hospital-diagnosed pneumonia	Beta	28.00%	9.95%	[15]
All-cause AOM (3 + 1)	Beta	19.00%	6.89%	[16]
Vaccine serotype coverage in Bhutan				
PCV7	Beta	31.58%	10.39%	RCDC
PCV10	Beta	47.37%	11.16%	RCDC
PCV13	Beta	57.89%	11.04%	RCDC
PCV7 serotype coverage in US				[17]
%Coverage in aged 10–39	Fixed	71.30%		
%Coverage in aged 40–64	Fixed	65.40%		
%Coverage in aged ≥65	Fixed	69.70%		
%IPD fall among unvaccinated population in US				[18]
%Fall in aged 20–39	Beta	40.00%	4.59%	
% Fall in aged 40–64	Beta	14.00%	4.59%	
% Fall in aged ≥65	Beta	29.00%	3.57%	
Probability of developing sequelae				
Epilepsy after pneumococcal meningitis	Beta	10.34%	5.56%	[9]
Hearing loss after pneumococcal meningitis	Beta	3.45%	3.33%	[9]
Neurodevelopmental impairment^c^ after pneumococcal meningitis	Beta	34.48%	8.68%	[9]
Hearing loss after AOM	Beta	5.10%	0.06%	[6]
Case fatality rates				
All-cause pneumonia	Beta	0.60%	0.07%	[4]
Pneumococcal meningitis	Beta	7.74%	1.43%	[4]
Pneumococcal bacteraemia	Beta	9.52%	3.67%	[9]
Direct medical costs (USD)				VPDP
PCV10 cost per dose	Fixed	3.05		
PCV13 cost per dose	Fixed	3.55		
Programme delivery cost per dose	Fixed	3.74		
Treatment cost per episode				Primary data collection
Pneumococcal meningitis aged <1	Beta	394	157	
Pneumococcal meningitis aged 1–4	Beta	400	159	
Pneumococcal meningitis aged 5–14	Beta	410	168	
Pneumococcal meningitis aged ≥15	Beta	433	198	
Pneumococcal bacteremia aged <1	Beta	242	143	
Pneumococcal bacteremia aged 1–4	Beta	248	149	
Pneumococcal bacteremia aged 5–14	Beta	260	142	
Pneumococcal bacteremia aged ≥15	Beta	315	162	
Hospitalized pneumonia aged <1	Beta	82	19	
Hospitalized pneumonia aged 1–4	Beta	83	19	
Hospitalized pneumonia aged 5–14	Beta	83	20	
Hospitalized pneumonia aged ≥15	Beta	122	11	
Non-hospitalized pneumonia aged <1	Beta	9	3	
Non-hospitalized pneumonia aged 1–4	Beta	9	3	
Non-hospitalized pneumonia aged 5–14	Beta	9	3	
Non-hospitalized pneumonia aged ≥15	Beta	21	1	
AOM aged <1	Beta	9	2	
AOM aged 1–4	Beta	9	2	
AOM aged 5–14	Beta	9	2	
AOM aged ≥15	Beta	4	2	
Treatment cost per year				Primary data collection
Epilepsy aged <1	Beta	123	46	
Epilepsy aged 1–4	Beta	122	45	
Epilepsy aged 5–14	Beta	145	53	
Epilepsy aged ≥15	Beta	170	64	
Hearing loss aged <1	Beta	129	112	
Hearing loss aged 1–4	Beta	129	112	
Hearing loss aged 5–14	Beta	129	112	
Hearing loss aged ≥15	Beta	129	112	
Neurodevelopment impairment aged <1	Beta	191	58	
Neurodevelopment impairment aged 1–4	Beta	191	58	
Neurodevelopment impairment aged 5–14	Beta	191	58	
Neurodevelopment impairment aged ≥15	Beta	189	55	

Adjusted utilities for annual cycle (using HUI3)				[19]
Pneumococcal meningitis (utility = 0.34, for 20 days)	Beta	0.9638	0.0046	
Pneumococcal bacteraemia (utility = 0.55, for 12 days)	Beta	0.9852	0.0025	
Pneumonia (utility = 0.59, for 8 days)	Beta	0.9910	0.0020	
AOM (utility = 0.71, for 2 days)	Beta	0.9984	0.0001	
Epilepsy	Beta	0.6400	0.0738	
Hearing loss	Beta	0.5500	0.0554	
Mild mental retardation	Beta	0.6900	0.0707	
Severe mental retardation	Beta	0.1000	0.1085	
Mental retardation + epilepsy	Gamma	0.0001	0.0943	

AOM: acute otitis media. IPD: invasive pneumococcal disease. RCDC: Royal Centre for Disease Control (http://www.rcdc.gov.bt/WEB/). VPDP: Vaccine Preventable Disease Programme, Department of Public Health.

a All-cause meningitis incidence [4] x Proportion of meningitis caused by *S. pneumoniae* (26.1% in child [7], 11.6% in adults [8]).

b PCV13 values were derived based on PCV7 data. See details in the Supplementary Table 1.

c Neurodevelopmental impairment included mild mental retardation, severe mental retardation and mental retardation with epilepsy.

#### 2.2.2 Direct vaccine effects (vaccine efficacy)

The vaccine coverage was assumed to be 97% based on the nationally reported coverage data of pentavalent vaccine which is given to children at the same schedule [20]. The data on serotypes coverage among the positive samples were locally collected from the clinical laboratory, Jigme Dorji Wangchuck National Referral Hospital (JDWNRH). The population serotype coverage rate for PCV7, PCV10, and PCV13 were 32%, 47.4%, and 58%, respectively as shown in Supplementary Fig. 2.

The vaccine efficacy of the 2 + 1 schedule of PCV10 was derived from three randomized clinical trials [14], [15], [16]. The vaccine efficacy of PCV13 was extrapolated using the efficacy of PCV7 [12], [13] and serotype coverage of PCVs in Bhutan as can be seen from Supplementary Table 2. Moreover, a prior study found that the efficacy against vaccine-type invasive pneumococcal disease for the 2 + 1 and 3 + 1 schedules of PCV10 were 92% and 100%, respectively [14], therefore, an 8% reduction of vaccine efficacy for the 2 + 1 schedule compared to the 3 + 1 schedule was assumed. The overall efficacy for PCV10 against invasive pneumococcal disease (44%) was calculated by multiplying PCV10 vaccine-type efficacy against invasive pneumococcal disease (92%) with the local serotype coverage of PCV10 (47.4%). The overall PCV13 efficacy against invasive pneumococcal disease (47%) was derived by multiplying the PCV7 vaccine-type efficacy against IPD (89%) with the local serotype coverage of PCV13 (57.9%) and multiplying with 8% efficacy reduction.

#### 2.2.3 Indirect vaccine effects (herd protection)

This study assessed the indirect effects (herd protection) against invasive pneumococcal disease among unvaccinated populations. Herd protection refers to the population-level effects of mass vaccination on unvaccinated persons. Proportion of vaccinated population must be more than 80% (as a threshold) in order to acquire the herd protection [21], [22]. Therefore, the indirect effects of PCVs in Bhutan can occur because it is very likely that the vaccination coverage in Bhutan will be higher than the threshold if introduced. The percentage change in invasive pneumococcal disease infections among unvaccinated individuals in Bhutan was estimated using the percentage reduction of invasive pneumococcal disease incidence in the United States after the introduction of PCV7 [17], and adjusting for the difference of country-specific vaccine serotype [18]. The duration of vaccine protection was assumed to be 8.3 years in accordance with a previous study [23] for both direct and indirect vaccine effects.

#### 2.2.4 Costs and outcomes

Direct medical costs were calculated in USD in 2017, comprising vaccine delivery costs and treatment costs. The data were collected from six health facilities in the country, representing each region. The health care providers were interviewed about their consultation time and resources used for the treatment. Unit costs of drugs and consumables were collected for 2016–2017 quoted prices from the Medical Supplies & Procurement Division in Bhutan. The estimated costs of introducing PCV10 and PCV13 included cold chain equipment, vaccine costs, transportation, training, information, education, communication, and monitoring and evaluation (Supplementary Table 2).

Health outcomes were measured in terms of QALY gained, number of pneumococcal episodes averted, and number of deaths prevented among the vaccinated and unvaccinated populations. QALYs were determined through multiplying the life expectancy in year with the utility value. For infectious diseases, utility scores change over the year, hence, the fraction of number of illness days were used to calculate utility over the full annual cycle before estimating QALYs. For example, 20 days for meningitis with a lower utility score of 0.34, and a utility score of 1.00 for the 345 days, which resulting an adjusted score of 0.96 was applied for the entire year instead. The Thai study reported the utilities measured by using four instruments, and suggested to use the HUI3 for measuring utility of sensory impairment [19]. As this study included this health condition, therefore, the utility scores measuring by the HUI3 were used.

### 2.3 Data analysis

#### 2.3.1 Incremental cost-effectiveness ratio

Cost-effectiveness results were presented as ICER per QALY gained. The ICER was calculated using the formula:

$$\mathrm{ICER}=\frac{\mathrm{Cost}\;\mathrm{of}\;\mathrm{intervention}-\mathrm{Cost}\;\mathrm{of}\;\mathrm{comparator}}{\mathrm{QALY}\;\mathrm{of}\;\mathrm{intervention}-\mathrm{QALY}\;\mathrm{of}\;\mathrm{comparator}}$$

Based on discussions during a stakeholder consultation meeting on 26 June 2017, a cost-effectiveness threshold of 1xGPD per capita or USD 2708 per QALY gained was deemed to be appropriate for this study.

#### 2.3.2 Sensitivity analysis

In order to determine the level of uncertainty for all parameters, a probabilistic sensitivity analysis was performed with 1000 iterations of Monte-Carlo simulation to yield a range of possible values for costs and QALYs. The gamma distribution was used when parameter values ranged between zero and infinity. The beta distribution was used for parameters which had values that ranged between zero and one. However, mental retardation + epilepsy utility has a relatively low mean score (0.0001) and a wide variation (SE = 0.0943), resulting a lower limit is less than zero. Under this circumstance beta distribution is inappropriate. Gamma distribution, an alternative approach, is recommended in this case [24]. Therefore, we do the transformation X = 1-U (utility decrement) and fit gamma distribution to X. A one-way sensitivity analysis was conducted to determine the uncertainty of results caused by each parameter individually. The plausible range for each parameter was examined based on its 95% confidence interval. The impacts of discount rate (0% and 6%), duration of vaccine protection (5 and 10 years), and vaccine costs were also assessed.

#### 2.3.3 Budget impact analysis

A budget impact analysis was carried out to forecast the financial implications over a period of five years to implement PCV10 or PCV13. A total of 14,432 births were reported in 2016 [4] and the study assumed a constant birth rate for the next five years. Additionally, costs related to unvaccinated populations who could be influenced by indirect benefits of the vaccine policy (i.e. older children, adults, and elderly) were included in the analysis. The budget was forecasted for both vaccinated and unvaccinated populations and was categorized into vaccination and treatment budgets.

#### 2.3.4 Human resources for health

The workloads, due to PCVs implementation, of various categories of health workers were estimated using the methods from a prior study [25]. We identified the set of healthcare services, the number of episodes for each disease, and the tasks and consultation time needed to provide the services. The number of human resource for health required for the implementation of the PCVs and the treatment of pneumococcal-related diseases were calculated in terms of full-time equivalent (FTE) which is equal to one employee that works on a full-time basis. In Bhutan, healthcare workers work for six days a week with 20 national holidays in a year. As a result, 293 workdays were identified annually. The average working hour per day is six hours. Therefore, the FTE is calculated through net working days in a year × working hours per day × 60 min. The total working time per year or one FTE corresponded to be 105,480 min/person/year.

### 2.4 Ethical clearance

The ethical clearance was approved by the Research Ethics Board of Health, Royal Government of Bhutan, Ministry of Health with reference number REBH/PO/2016/092.

## 3 Results

Compared to a no vaccination scenario, the introduction of PCV13 into routine immunization programme could avert 2916 and 261 pneumococcal episodes in vaccinated and unvaccinated populations, respectively, while if PCV10 is introduced, a total of 2003 pneumococcal episodes in a vaccinated population and 214 pneumococcal episodes in an unvaccinated population could be averted (Supplementary Fig. 1). PCV13 could prevent 30 deaths and PCV10 could prevent 18 deaths in a vaccinated population while the same vaccines could prevent 12 and 10 deaths in an unvaccinated population, respectively.

**Fig. 2:**
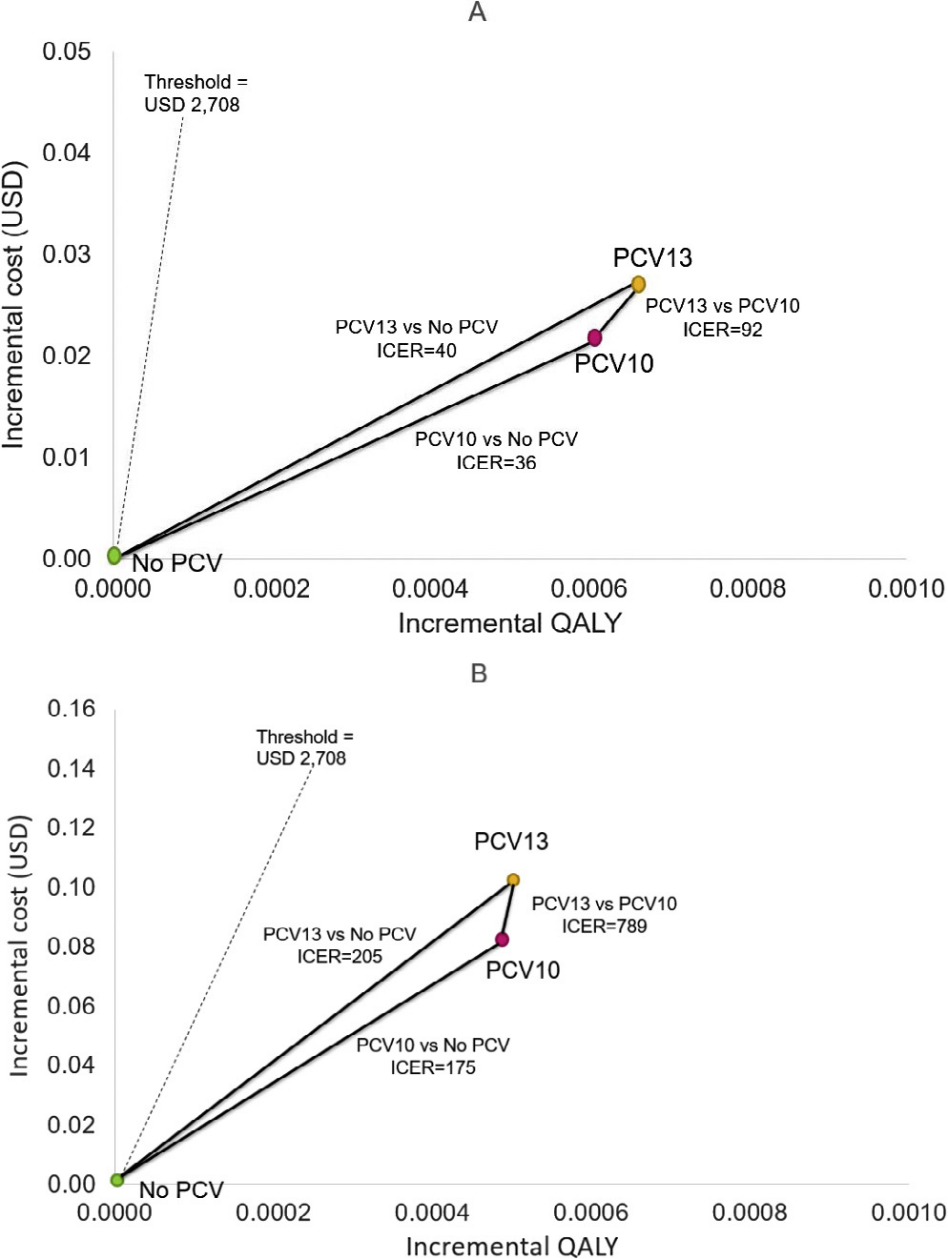
Cost-effectiveness plane: Threshold at USD 2708.

Fig. 2 shows the ICERs of PCVs. Including indirect vaccine effects, PCV10 and PCV13 gained 0.0006 and 0.0007 QALYs with additional lifetime costs of USD 0.02 and USD 0.03 per person, respectively, compared to no vaccination (Fig. 2A). The ICERs per QALY gained of PCV10 and PCV13 were USD 36 and USD 40 per QALY gained, respectively. The ICER of PCV13 versus PCV10 was found to be USD 92 per QALY gained. Without indirect vaccine effects, the ICERs per QALY gained of PCV10 and PCV13 were USD 175 and USD 205, respectively, compared to no vaccination (Fig. 2B).

**Fig. 3:**
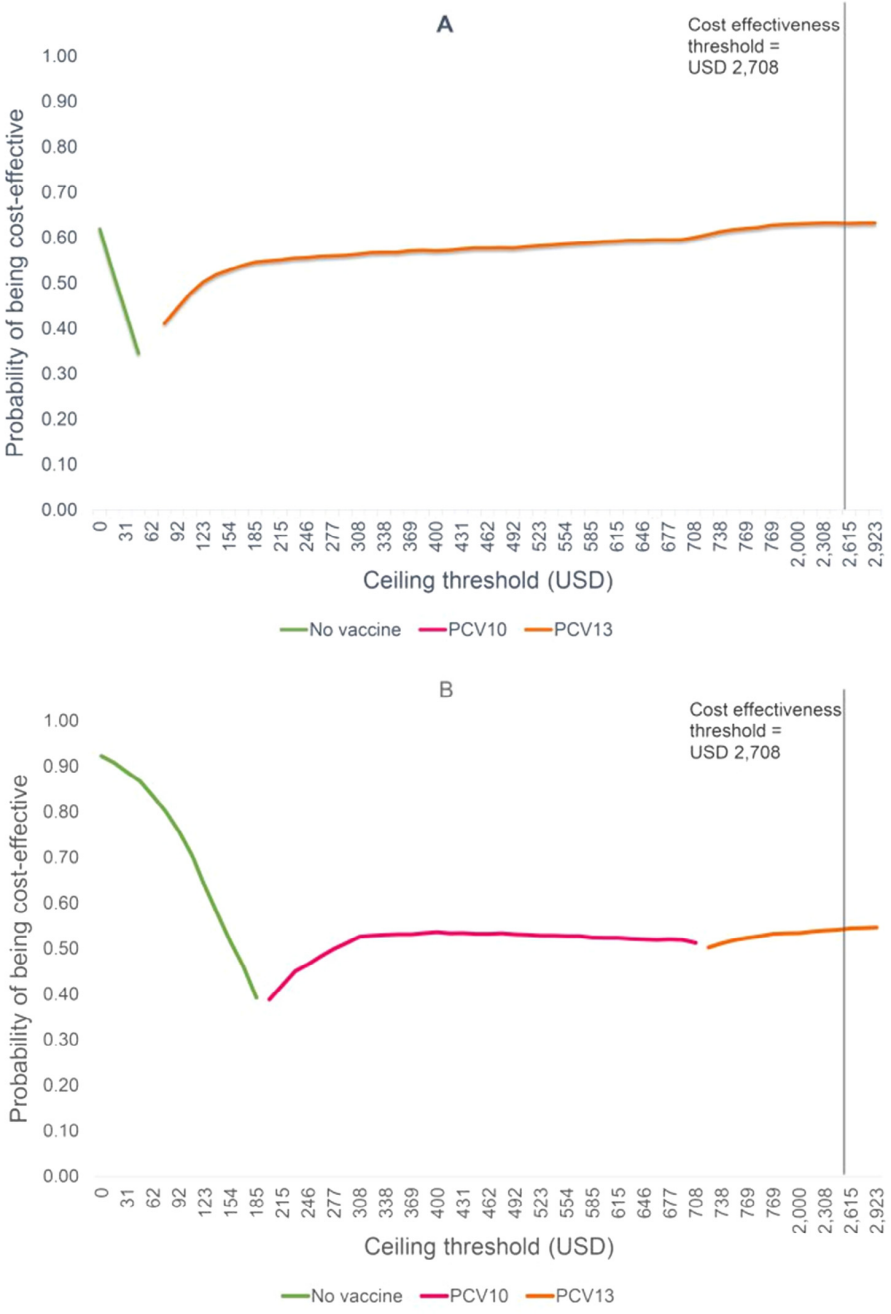
Cost-effectiveness acceptability frontier.

Fig. 3 illustrates the cost-effectiveness acceptability frontier examining the optimal PCV strategy across a range of cost-effective threshold. When considering indirect vaccine effects, PCV13 was the optimal strategy at any threshold greater than USD 77 (Fig. 3A). Without indirect effects, at any threshold lower than USD 200, no vaccination was the most favourable option (Fig. 3B). However, at thresholds between USD 200 and 708, PCV10 was the optimal option. Furthermore, at threshold exceeded USD 723, PCV13 became the most favourable option.

The findings of the study indicate that both the vaccines are cost-effective at the Gavi’s prices; USD 3.05 of PCV10 per dose and USD 3.55 of PCV13 per dose. Our analysis indicates that the maximum prices for these two vaccines to be cost-effective are USD 7.95 for PCV10 and USD 8.65 for PCV13 (Supplementary Fig. 2). Moreover, the variation in serotype coverage, duration of vaccine protection, excluding indirect vaccine effects (herd protection), and discount rate had a large impact on the ICER, however, these parameters do not cause the ICER to exceed the cost-effectiveness threshold of USD 2708 per QALY gained (Supplementary Fig. 3).

**Fig. 4:**
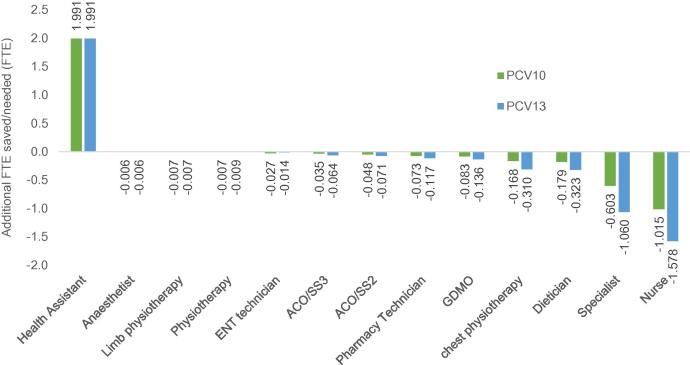
Impact on human resource for health care.

When implementing PCVs into the Extended Programme for Immunization, the FTE of one health assistant would increase by 2 per year while the FTE of other health workers would decrease, particularly for specialists (from 0.6 to 1.1 FTE) and nurses (from 1 to 1.6 FTE) as seen in Fig. 4. The budget impact analysis for the five-year period revealed that the treatment cost would decline by 8.5% and 13.6% considering the inclusion of PCV10 and PCV13, respectively. However, the total budgetary requirement is anticipated to increase approximately to USD 3.77 million for PCV10 and USD 3.75 million for PCV13 (Table 2).

**Table 2:** Budget impact analysis in five years (thousand USD).

Year	No vaccine			PCV10			PCV13		
	Treatment	Vaccine	Total	Treatment	Vaccine	Total	Treatment	Vaccine	Total
1	585	–	585	548	294	842	532	315	848
2	591	–	591	545	148	692	525	169	694
3	631	–	631	572	148	720	549	169	718
4	666	–	666	595	148	743	568	169	737
5	705	–	705	622	148	769	589	169	758
Total	3175	–	3175	2883	883	3766	2762	992	3754
Incremental budget	–	–	–	–	–	591	–	–	578

## 4 Discussion

Introducing pneumococcal conjugate vaccines at their current price offered to Gavi into the routine immunization programme in Bhutan would be cost-effective. Our analysis found that PCV13 would be a preferable choice as it would yield better health outcomes, in terms of episodes of pneumococcal disease and number of deaths, and would incur a lower five-year budget.

Both PCV10 and PCV13 are cost-effective at the threshold of USD 2708 per QALY gained. Even though PCV10 produces a slightly lower ICER than PCV10, PCV13 yields a better outcome than PCV10, a result similar to studies conducted in Paraguay and Thailand [6], [26]. However, PCV13 requires a lower budget compared to PCV10. This can be explained by the impact of the time horizon and costs per-dose of the two vaccines. If a time horizon of less than seven years was applied, PCV13 would yield a lower ICER than PCV10. Additionally, PCV13 is 17% more costly than PCV10. If the cost of PCV13 was reduced by 5% from the current cost, PCV13 would produce a similar ICER as PCV10.

The introduction of pneumococcal conjugate vaccines in Bhutan would not only prevent pneumococcal related illnesses, reduce treatment costs and deaths among both vaccinated and unvaccinated populations, but it would also significantly reduce the workload of healthcare workers, especially specialists and nurses. However, the introduction of these vaccines would increase the workload of health assistants who are primarily involved in the vaccination programme by two FTE. As there is only handful of paediatricians in the country, reducing one FTE of a paediatrician would be very significant.

Until now there have been at least 20 economic evaluation studies conducted in Asia [6], [27], [28], [29], [30], [31], [32], [33], [34], [35], [36], [37], [38], [39], [40], [41], [42], [43], [44]. However, this is the first study conducted in South Asia. The findings from this study are similar to many other previous assessments in terms of confirming the value for money of pneumococcal conjugate vaccines among children.

Unlike other studies, this is the first study that comprehensively addresses other impacts of vaccine introduction including five-year budget, vaccine price threshold, and human-resources for health required or saved. This additional information, apart from the cost-effectiveness aspect, is very important to low and lower-middle income countries such as Bhutan because value for money is not the only criterion for health resource allocation. Financial sustainability and feasibility are always key decision-making criteria because the country is facing financial challenges to pay for new vaccines due to the fact that Bhutan has already graduated from Gavi, the Vaccine Alliance. In addition, because Bhutan is a relatively small country with a small health workforce, it is very important to estimate the impact on its health workforce to ensure the feasibility of new policies that are introduced.

There are some limitations to this study which should be acknowledged. Firstly, the data on sequelae due to pneumococcal diseases and the health utility estimates were transferred from studies conducted in Thailand due to unavailability of local data. Likewise, the data on herd protection was adapted from the United States of America. Secondly, the incidence of outpatient visits in Bhutan's National Referral Hospital was based on the data collected between January and March 2017 and this may not capture seasonal variations, if they exist. Thirdly, we adopted a government perspective; therefore, we did not consider the direct non-medical costs borne by households including traveling costs for seeking care, and productivity loss of caregivers. If included, the vaccines are likely to be more cost-effective in Bhutan. Lastly, this study does not consider the serotype replacement. Since, there has been reported that non-vaccine serotypes have increased after the widespread use of PCV7, in particular 19A (included in PCV13 but not in PCV10) [45], [46], [47]. However, in South Korea, an increasing of 19A was found before routine use of PCV7 [48]. There have been arguments that increasing in non PCV7 serotypes among some populations might be caused by other mechanisms, for example, immunosuppression and antibiotic use, but not serotype replacement [49]. Therefore, a rise of non-vaccine types in Bhutan is unpredictable. It is noteworthy that serotype replacement could be a factor in designing of PCV policies in Bhutan.

In conclusion, at the suggested threshold of 1xGDP per capita, which is equivalent to USD 2708, both vaccines are cost-effective, and we recommend that they be included in the routine immunization programme in Bhutan. Implementing PCV13 would avert more episodes of pneumococcal disease, save more lives, and incur a lower five-year budget compared to PCV10.

## Conflict of interest

None.
