# Supplementary material for: Towards the introduction of pneumococcal conjugate vaccines in Bhutan: A cost-utility analysis to determine the optimal policy option
Source: Vaccine. 2018 Mar 20;36(13):1757–65. doi: 10.1016/j.vaccine.2018.02.048 (PMC5858152; doi:10.1016/j.vaccine.2018.02.048)

Supplementary Figure 1: Predicted number of episodes and deaths averted for each vaccine and population type. (A) Episodes averted; (B) Deaths averted.

Supplementary Figure 2: Threshold analysis of PCV price per dose: at threshold of USD 2,708

Supplementary Figure 3: One-way sensitivity analysis.


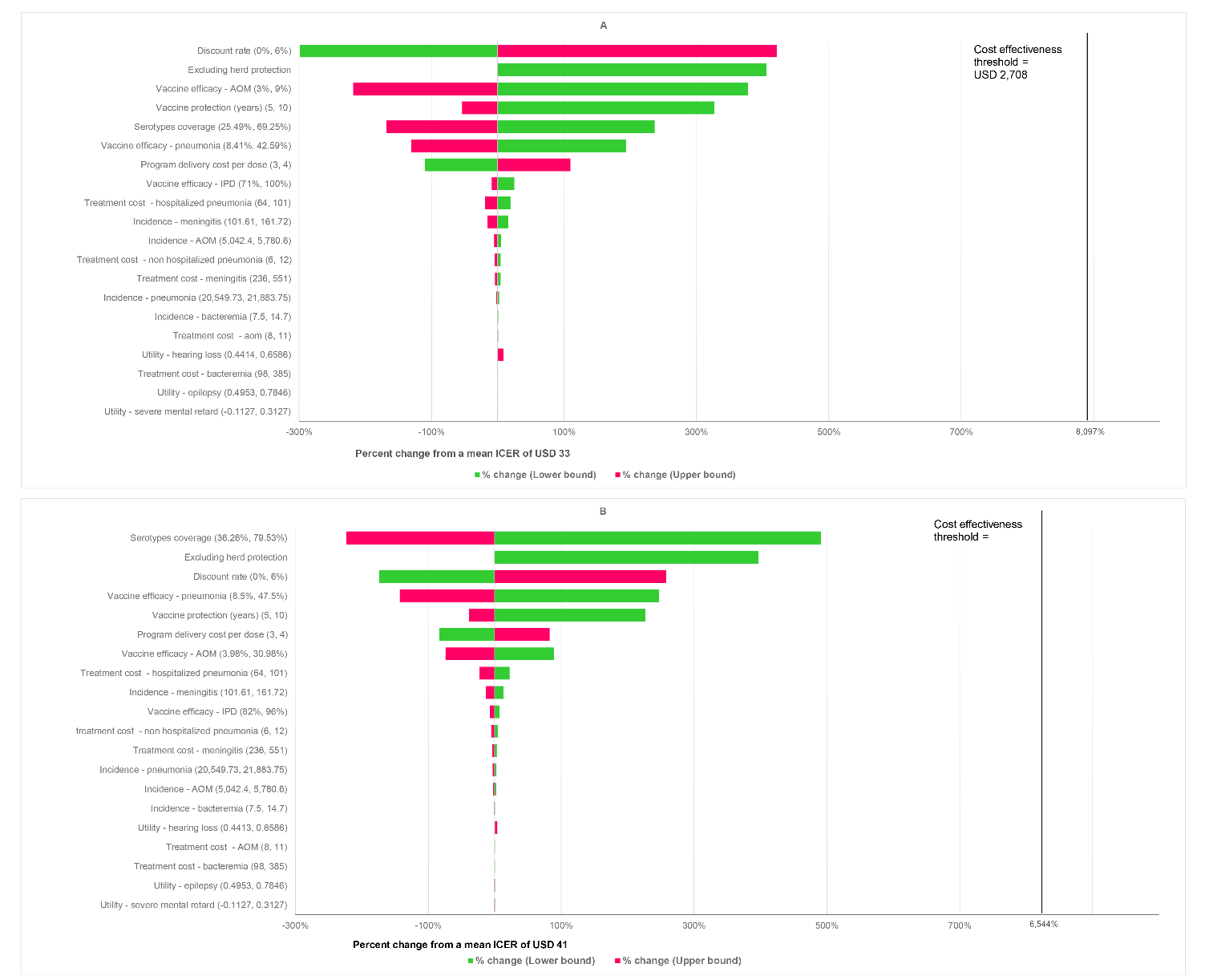

Supplement: Supplementary data 1 [file mmc1.docx]
