# Supplementary material for: Towards the introduction of pneumococcal conjugate vaccines in Bhutan: A cost-utility analysis to determine the optimal policy option
Source: Vaccine. 2018 Mar 20;36(13):1757–65. doi: 10.1016/j.vaccine.2018.02.048 (PMC5858152; doi:10.1016/j.vaccine.2018.02.048)
Supplement: Supplementary data 2 [file mmc2.docx]

Supplementary Table 1: PCV efficacies using schedule and serotype adjustment

|  | **PCV10** | **PCV13** |
| --- | --- | --- |
| All-type IPD | 44%^*^ | 47%^†^ |
| All-cause pneumonia | 28% | 43%^‡^ |
| All-cause AOM | 17%^§^ | 10%^¶^ |

^*^PCV10 efficacy against vaccine-type IPD x serotype coverage PCV10

^†^PCV7 (3+1) efficacy against vaccine-type IPD x 92% x serotype coverage PCV13

^‡^PCV7 (3+1) efficacy against pneumonia x 92% x serotype coverage PCV13/ serotype coverage PCV7

^§^PCV10 (3+1) efficacy against AOM x 92%

^¶^PCV7 (3+1) efficacy against AOM x 92% x serotype coverage PCV13/ serotype coverage PCV7

Supplementary Table 2: Vaccine introduction costs

| **No.** | **Cost category** | **Cost items** | **Estimated amount (USD)** |
| --- | --- | --- | --- |
| 1 | Cold chain equipment | - Refrigerators (28 Nos.) | 36,923 |
|  |  | - Cold boxes (10 Nos.) | 354 |
|  |  | - Vaccine carrier (100 Nos.) | 1,215 |
|  |  | - Freeze Alerts (200 Nos.) | 600 |
|  |  | - Digital Temperature recording (100Nos) | 923 |
|  |  | - Safety boxes (430 Nos. with 172 boxes at $15.25) | 262 |
| 2 | Vaccine cost | - PCV 10 (43,000 doses at $3.05) | 131,138 |
|  |  | - PCV13 (43,000 doses at $3.5/dose including wastage rate) | 152,815 |
|  |  | - AD Syringes (430 boxes with 100 syringes at $5) | 2,169 |
| 3 | Transportation | - Fuel for vehicle | 1,538 |
|  |  | - DSA/TA EPI In charges (6 persons) | 10,769 |
|  |  | - TA/DA for HA/Care takers (To collect vaccine from the district Hospitals) | 1,846 |
| 4 | Training | - Training of Trainers | 9,231 |
|  |  | - District Level training | 46,154 |
|  |  | - Development of guidelines/manual | 4,615 |
|  |  | - Printing of manual | 6,154 |
| 5 | Information/education & Communication (IEC) | - Development of IEC materials | 4,615 |
|  |  | - Dissemination/airing through mass media (TV/Radio/Print) | 3,077 |
|  |  | - Printing of posters/pamphlets | 1,538 |
|  |  | - High level advocacy/sensitization | 2,308 |
| 6 | Monitoring and evaluation (M&E) | - National level supervisors to monitor during introduction period | 7,692 |
|  |  | - District level supervision and monitoring | 15,385 |
|  |  | - Documentation and Report writing | 3,077 |
|  | **Total (USD)** |  | 313,262 |

Nos: numbers, AD: Auto-Disable, DSA: daily sustenance allowance, TA: travel allowance, DA: daily allowance, HA: Health Assistant

Supplementary Table 3: Budget impact analysis in five years (thousand USD). A: vaccinated population, B: unvaccinated population

A: Vaccinated population

| **Year** | **No vaccine** | | | | **PCV10** | | | **PCV13** | | |
| --- | --- | --- | --- | --- | --- | --- | --- | --- | --- | --- |
|  | **Treatment** | **Vaccine** | **Total** | **Treatment** | | **Vaccine** | **Total** | **Treatment** | **Vaccine** | **Total** |
| 1 | 123 | - | 123 | 89 | | 294 | 383 | 74 | 316 | 389 |
| 2 | 163 | - | 163 | 121 | | 147 | 268 | 102 | 169 | 271 |
| 3 | 212 | - | 212 | 158 | | 147 | 305 | 135 | 169 | 304 |
| 4 | 263 | - | 263 | 197 | | 147 | 344 | 169 | 169 | 339 |
| 5 | 316 | - | 316 | 238 | | 147 | 385 | 206 | 169 | 375 |
| Total | 1,077 | - | 1,077 | 803 | | 883 | 1,686 | 686 | 992 | 1,678 |
| **Incremental budget** | **-** | **-** | **-** | **-** | | **-** | **608** | **-** | **-** | **601** |

B: Unvaccinated population

| **Year** | **No Vaccine** | | | | **PCV10** | | | **PCV13** | | |
| --- | --- | --- | --- | --- | --- | --- | --- | --- | --- | --- |
|  | **Treatment** | **Vaccine** | **Total** | **Treatment** | | **Vaccine** | **Total** | **Treatment** | **Vaccine** | **Total** |
| 1 | 462 | - | 462 | 459 | | - | 459 | 458 | - | 458 |
| 2 | 427 | - | 427 | 424 | | - | 424 | 423 | - | 423 |
| 3 | 418 | - | 418 | 415 | | - | 415 | 414 | - | 414 |
| 4 | 403 | - | 403 | 399 | | - | 399 | 398 | - | 398 |
| 5 | 388 | - | 388 | 384 | | - | 384 | 383 | - | 383 |
| Total | 2,098 | - | 2,098 | 2,080 | | - | 2,080 | 2,076 | - | 2,076 |
| **Incremental budget** | **-** | **-** | **-** | **-** | | **-** | **-18** | **-** | **-** | **-22** |
